# SARS-CoV-2: Origin, Intermediate Host and Allergenicity Features and Hypotheses

**DOI:** 10.3390/healthcare9091132

**Published:** 2021-08-30

**Authors:** Yuyi Huang, Junmou Xie, Yuhe Guo, Weimin Sun, Ying He, Kequn Liu, Jie Yan, Ailin Tao, Nanshan Zhong

**Affiliations:** 1 The Second Affiliated Hospital, The State Key Laboratory of Respiratory Disease, Guangdong Provincial Key Laboratory of Allergy & Clinical Immunology, Guangzhou Medical University, Guangzhou 510260, China; huangyuyi@gzhmu.edu.cn (Y.H.); xiejunmou@126.com (J.X.); guoyuhe2013@126.com (Y.G.); sumyj1000@163.com (W.S.); heying0605@163.com (Y.H.); 2 Wuhan Regional Climate Center, Wuhan 430074, China; 3 The State Key Laboratory of Respiratory Disease, National Clinical Research Center for Respiratory Disease, Guangzhou Institute of Respiratory Health, The First Affiliated Hospital of Guangzhou Medical University, Guangzhou 510120, China

**Keywords:** SARS-CoV-2, spike protein, intermediary reservoir, allergenicity, MHC binding affinity, self-limitation, spontaneous mutation, early immunization

## Abstract

The goal of this study is to investigate the probable intermediate hosts and the allergenicity of the notorious virus SARS-CoV-2 to understand how this virus emerged. The phylogenetic analysis of the virus spike proteins indicates that SARS-CoV-2 falls into various small subclades that include a bat coronavirus RaTG13, suggesting bats as a likely natural origin. Refined alignment of the spike protein in NCBI found several fragments that are specific to SARS-CoV-2 and/or SARS-CoV are specific to *Rattus norvegicus* and/or *Mus musculus*, suggesting that rodents are the intermediate reservoir of SARS-CoV-2 and SARS-CoV. To evaluate the allergenicity values, the binding affinities of human leukocyte antigen (HLA) class I or II molecules with the spike proteins were calculated, and the results showed that both SARS-CoV-2 and SARS-CoV are predicted to bind to fourteen HLA class I and II molecules with super-high HLA allele-peptide affinities. The infection rate of individuals who have HLA alleles with very high binding affinities who might become infected and develop into refractory patients if there were no medical or non-medical interventions is about 7.36% and 4.78% of Chinese and Americans, respectively. Extremely high temperature and exceptionally low precipitation, the common climate factors between the outbreak sites of COVID-19 in Wuhan in 2019 and SARS in Guangdong in 2002, might have promoted coronavirus evolution into more virulent forms. Our hypothesis suggests that early immunization with an allergenically-engineered virus, in combination with continued surveillance of meteorological factors and viral mutations, may be one of the most powerful prophylactic modalities to fight this virus.

## 1. Introduction

The recent outbreak of COVID-19 across the whole world was caused by a novel beta coronavirus isoform which was designated as SARS-CoV-2 by the International Committee on Taxonomy of Viruses (ICTV) based on the sequence of the viral RNA genome. The World Health Organization (WHO) claimed an international public health emergency for the outbreak in January and, later, a global pandemic in March 2020. As of 22 March 2021, more than 25,794,100 infected cases were reported in over 200 countries and regions. This prompted scientists to identify how the epidemic affected such a substantial amount of people in the world. Coronaviruses are known to circulate in birds and mammals, including bats [1]. Several publications have recently explored the origin of SARS-CoV-2. Based on genomic sequence analysis, Shi and colleagues demonstrated that the novel virus is 96% homologous to a Yunan bat coronavirus at the whole-genome level [2], while Wu et al. reported only 89.1% nucleotide similarity between the virus and other SARS-like coronaviruses (*Betacoronavirus sarbecovirus*) originally found in bats in China [3]. Guo et al. further demonstrated that bats and minks are two likely candidate reservoirs of the novel virus [4].

It is critical to identify the immediate reservoirs of SARS-CoV-2 and how the virus is passed onto humans. Recently reported results have been controversial. The results from Wei et al. suggested that snakes are the most probable wild animal reservoir for the virus, based on their relatively synonymous codon usage bias compared to other animal species [5]. Work from Guan and colleagues suggested that pangolins (*Manis javanica*) should be considered as a possible intermediate host for the novel coronavirus based on the 85.5% to 92.4% similarity of the viruses found in pangolins to a partial length (~86.3%) of the SARS-CoV-2 genome sequence [6]. Two other studies implied that cats may be involved in virus infection and transmission [7,8]. SARS-CoV-2 is a single-stranded RNA coronavirus bearing a high frequency of RNA recombination, and the stability of the virus would be affected by environmental conditions, such as temperature, humidity, atmospheric pressure, etc. [9,10,11]. How to keep codon-based evolution analysis of this virus accurate is therefore a perplexing and challenging question.

Allergenicity is referred to as the ability of an antigen to induce an aberrant or detrimental immune response in the host, which is an overreaction and different from a normal immune response in that it does not result in a protective/prophylactic effect but instead causes physiological dysfunction and/or tissue damage [12]. In the early phase of an allergic reaction, antigens are presented through major histocompatibility complex (MHC) in vertebrates and HLA (human leukocyte antigen) in humans to T cells to activate adaptive immunity [13,14,15,16,17]. Whether an antigen is able to be presented to T cells or not depends on the binding affinity of that antigen with MHC/HLA molecules of antigen-presenting cells (APCs). The stronger the binding affinity, the more likely the antigen would be presented outside of APCs, and thus, the higher the allergenicity. Such an antigen is more likely to trigger danger signals and activate downstream inflammatory pathways and cytokine storms [12,18]. Therefore, the binding affinity of antigens to HLA molecules is a key indicator of the allergenicity and the presentation potency of those antigens. Immunologically, proteins from SARS-CoV-2 should also be presented as antigens by APCs as they touch human bodies. High allergenicity of a viral antigen typically elicits a rapid elevation of various inflammatory factors, and often renders viral antigens liable to induce cytokine storms [19]. Profilin is a panallergen and exhibits a configuration of *α*-*β*-*α* layers, a similar structure component element shared by different allergens [20]. It can induce only mild symptoms like oral allergy syndrome in the allergic population [21,22]. Therefore, the highest values of profilin binding affinity to HLA I (≥0.9) and HLA II (≥0.8) molecules will be cited as the lowest cutoff values to discriminate the binding affinity of different HLA molecules to the spike protein of SARS-CoV-2 and/or SARS-CoV.

This study aims to: (1) Determine the potential virus intermediate reservoir by carrying out comprehensive amino acid sequence analysis and comparison of sliding sequence fragments of the novel virus with all sequences from mammals available in the NCBI database; (2) Analyze the allergenicity of the spike protein in SARS-CoV-2 and compare it with that of SARS-CoV to explain the mechanism of the COVID-19 pandemic from a new perspective. Furthermore, we systematically compare climate data in the past 50 years to predict any relationship between the meteorological conditions and the survival/development of the virus.

## 2. Materials and Methods

### 2.1. Evolutionary Analysis

The amino acid sequences of the SARS-CoV spike glycoprotein derived from humans, civets and bats were downloaded from NCBI. Sequence alignment was performed to identify three functional subunits: receptor binding domain, N-terminal domain and coronavirus S2 glycoprotein. A phylogenetic tree was constructed using the maximum likelihood method with best protein models for different sequence groups using the MEGA7.0 program [23,24].

### 2.2. Key Sites Analysis

First, protein sequences of SARS-CoV-2 and SARS-CoV derived from humans, civets and bats were analyzed through multiple sequence alignment to locate all potential homologous sites. Then, a homology site screening program was constructed to classify these sites and to screen out the homologous key sites in accord with consistent sites among human SARS-CoV-2, SARS-CoV, and civet SARS-CoV, but 50–100% different from bat SARS-CoV.

### 2.3. Mouse Derived Peptide Analysis

The fixed-length sliding window method was used to split the spike glycoprotein sequence into equal length, non-overlapping peptide segments. Based on the latest version of the NCBI Reference protein library of whole organisms, a peptide source scanner was constructed to analyze the source of each peptide of the spike glycoprotein. The proportion of mouse-derived peptide was calculated, and the possible mouse-derived peptides were retained. By scanning in the NCBI Reference protein library and the non-redundant protein sequence library, Protein BLAST was performed to verify the exclusive origin of the peptides retained by the scanner.

### 2.4. Allergenicity Assessment and Infection Population Estimating

The allergenicity of SARS-CoV-2 and SARS-CoV was assessed by using the software NetMHC-4.0 [25,26] and NetMHCII-2.3 [27] to predict the binding affinity of human SARS-CoV-2 and SARS-CoV with HLA class I and class II molecules. Briefly, the sliding window approach was used to extract peptides from the full-length spike proteins of the viruses which were 20 and 9 amino acids in length for HLA Class II and Class I alleles, respectively. Fifty kinds of HLA class II alleles and 81 kinds of HLA class I alleles were selected for binding affinity prediction. The distributions of peptides of different binding affinities with different HLA class I and class II molecules were calculated. Based on allele frequencies in worldwide populations (http://www.allelefrequencies.net/, accessed on 27 August 2021) [28], the numbers of individuals that have alleles which tightly bind with the spike protein were predicted and the infection rates were calculated according to the Hardy–Weinberg equilibrium for the populations of China and the United States, respectively. Binding affinities of more than 0.9 to HLA class I molecules and more than 0.8 to HLA class II molecules were used as the cutoff values for superhigh allergenicity discrimination.

### 2.5. Meteorological Parameters Analysis

The monthly climate data of Wuhan and Guangdong from 1951 to 2019 were downloaded from the China Meteorological Network, including six climate characteristics, such as average temperature, average maximum temperature, average minimum temperature, precipitation, sunshine hours, and relative humidity (http://www.weather.com.cn/, accessed on 27 August 2021). Different meteorological factors, including 6 climate factors and 12 months from 1959 to 2019, were analyzed to obtain any clues regarding the outbreak of SARS-CoV-2. Using 12 months and 6 climatic features as primary data, 257,985 different combinations between month and climatic feature were produced. The correlation coefficients of different years under the combination of month and climatic features were calculated using R language. To construct a screening program aimed at 2019 and 2002, we screened out the climate combination features that exhibited a special strong correlation between the two years; that is, the corresponding climate combination features of Wuhan 2019 are very similar to those of Guangdong in 2002 (Pearson correlation coefficient > 0.8), but are less similar to those in most other years (Pearson correlation coefficient < 0.5). 

## 3. Results

### 3.1. Phylogenetic Analysis of SARS-CoV-2

**Figure 1:**
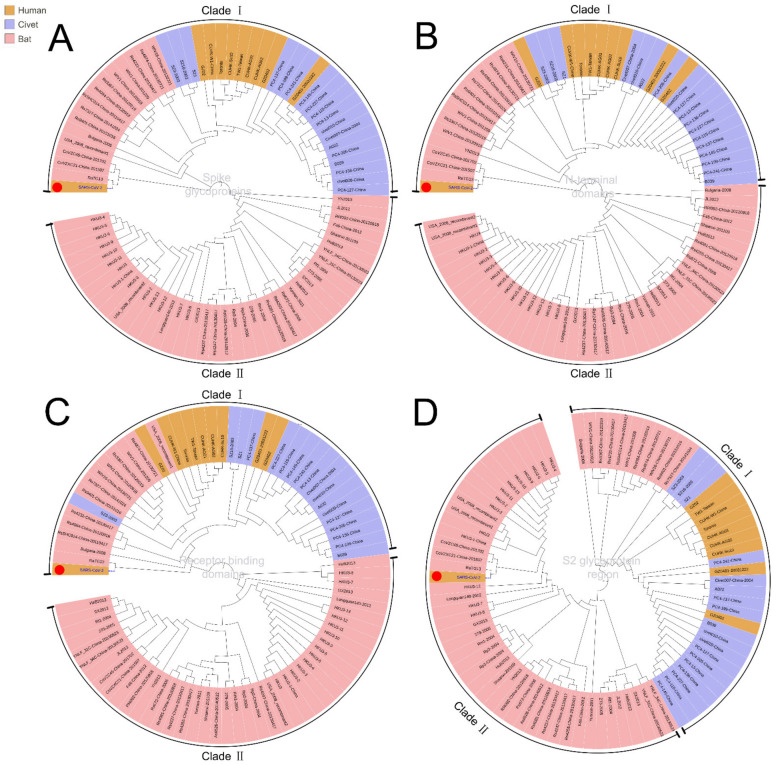
Phylogenic analysis of different domains in the spike glycoproteins of SARS-CoV-2, SARS-CoVs, and other beta-coronavirus of bat and civet origin using the maximum likelihood method. (**A**). Full-length spike glycoproteins; (**B**). The N-terminal domains; (**C**). Receptor binding domains; (**D**). S2 glycoprotein region; two clades were divided in all phylogenic trees. SARS-CoV-2, marked with a red dot, would fall into different subclades according to its different domains.

To trace the source of SARS-CoV-2 and its evolutionary path, we analyzed the evolutionary relationship of the spike glycoproteins of human SARS-CoV-2, SARS-CoV, and the coronaviruses reported in bat and civets (Figure 1A). The spike protein contains S1 and S2 domains. S1 contains the receptor-binding domain (RBD) and S2 mediates fusion with host membranes. Although there is a distinct evolutionary difference among human SARS-CoV-2, SARS-CoV and other coronaviruses, bat SARS-CoVs (including RaTK13, CoVZC45, CoVZXC21, etc.) have evolutionary proximity to human SARS-CoV-2. These bat SARS-CoVs are also located proximally to civet and human SARS-CoVs in the evolutionary tree (Figure 1, Clade I). These results suggest that the natural human SARS-CoV-2 most likely originated from bats. 

To demonstrate the evolutionary origin of human SARS-CoV-2 and other SARS-CoVs, we further analyzed the evolution of functional domains in spike glycoproteins. Phylogenetic results showed that the closest evolutionary relationship between SARS-CoV-2 and SARS-CoV mainly lies in the N-terminal and receptor binding domains (Figure 1A–D). However, the sequence in the S2 glycoprotein region of human SARS-CoV-2 is related more closely to that of bat SARS-CoV than human SARS-CoV (Figure 1D, Clade II), suggesting the S2 glycoprotein region is the likely cause for a pathogenetic difference between human SARS-CoV-2 and SARS-CoV. Therefore, the human SARS-CoV-2 is an independent branch located at the bottom of the evolutionary tree. 

### 3.2. Intermediate Host Analysis

**Figure 2:**
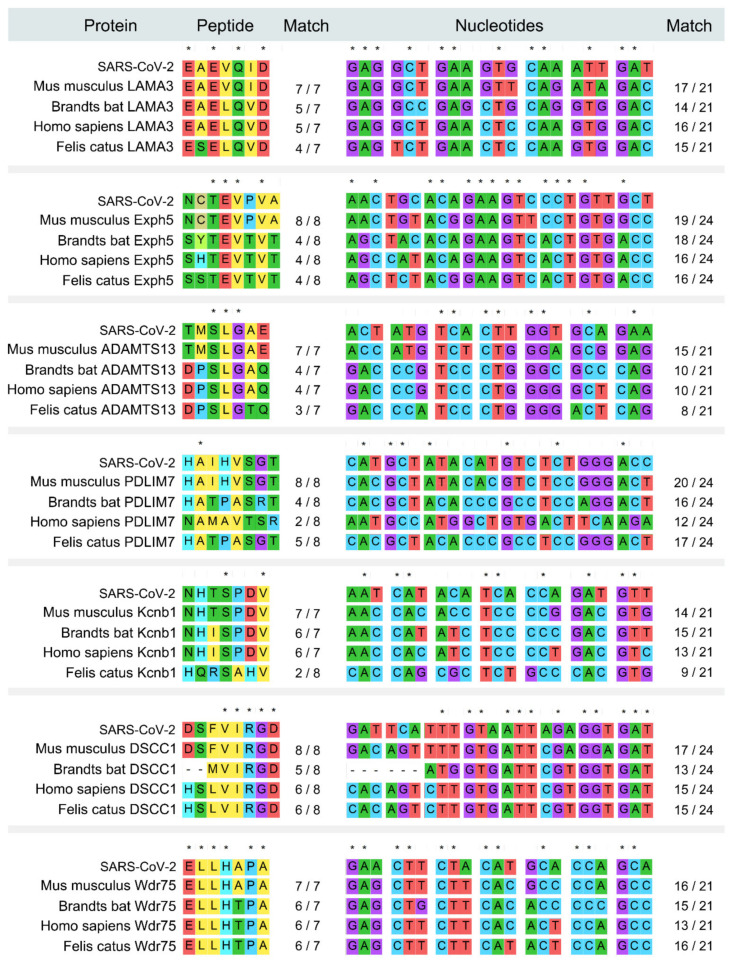
Alignments of the amino acid and nucleotide sequences from SARS-CoV-2, the rodent and other species.

To identify the intermediate hosts of SARS-CoV-2, we scanned and aligned the human SARS-CoV-2 spike glycoprotein in the entire biological database using the peptide sliding window approach. Seventeen mouse-derived peptide fragments exactly matched with peptides within the human SARS-CoV-2 spike glycoprotein, which contained a total of 118 amino acids of mouse origin (Table S1). We further verified these fragments in the NCBI BLAST reference protein library and the non-redundant protein sequences library, and the results showed that seven fragments are mouse-specific peptides and exist only in mouse databases (*Mus* or *Rattus*) but no other mammal databases (Figure 2), indicating that human SARS-CoV-2 harbors the peptide fragments common with those found in the mouse. Thus, the mouse could be the intermediate host of human SARS-CoV-2.

Fifteen mouse-derived peptide fragments were found in human SARS-CoV with an 100% match, which accounted for 105 amino acids of mouse origin (Table S2). Four of these fragments were verified as rat-specific peptides via NCBI BLAST (Table S2). 

The fragments EAEVQID and NHTSPDV are common mouse-derived peptides that exist in both SARS-CoV-2 and SARS-CoV (Table S1). Compared with SARS-CoV, human SARS-CoV-2 possesses more mouse-derived and mouse-specific peptides, indicating higher likelihood of mouse origin than SARS-CoV. There are two mouse-specific heterotopia peptides, NCTEVPVA(E) and ELLHAPA(H), that are uniquely identified in human SARS-CoV-2, but not in other SARS-CoVs derived from humans, civets or bats. Importantly, the mouse derived peptide TQRNFY found in human SARS-CoV-2 is also found in *Klebsella pneumonia*, which may be related to a potentially shared pathogenetic pathway. 

**Figure 3:**
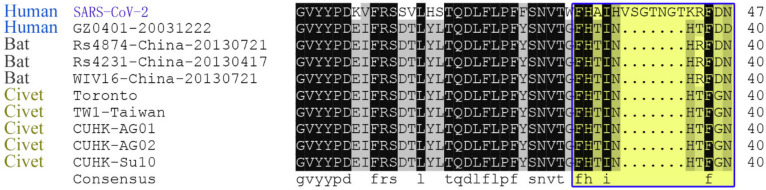
Alignment of the specific HAIHVSGT-containing region in the N-terminal domain of SARS-CoV-2 spike glycoprotein with that of human SARS-CoV and bat SARS-CoV.

**Figure 4:**
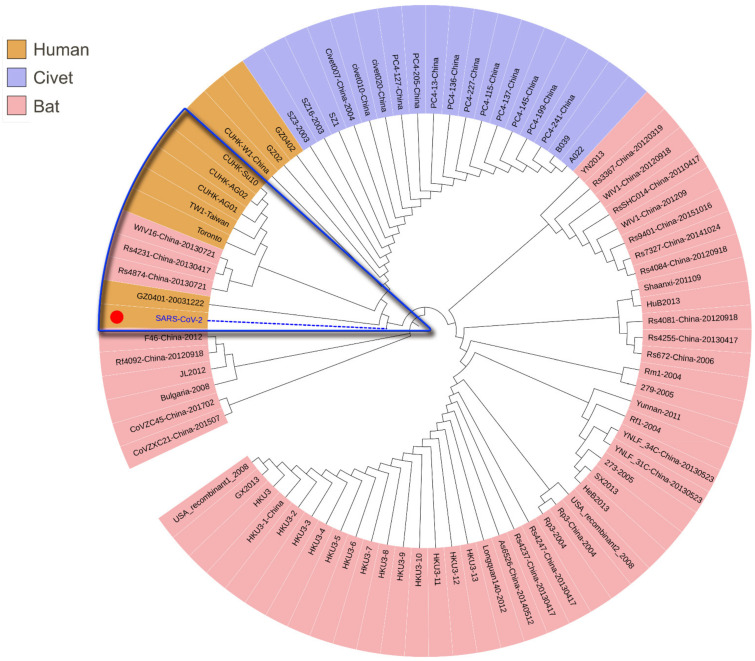
Phylogenic analysis by the maximum likelihood method for the specific region in the N-Table 2. SARS-CoVs. Sequences that have a close evolutionary relationship with SARS-CoV-2 were highlighted in the blue box.

Another fragment HAIHVSGT in the SARS-CoV-2 spike glycoprotein was found to be specifically identical in *Rattus norvegicus* and *Mus musculus* (Figure S1). In comparison with SARS-CoV spike glycoproteins derived from humans, civets and bats, this peptide is located in a specific insertion area of the N-terminal domain in the SARS-CoV-2 spike glycoprotein (Figure 3). We then extracted this specific HAIHVSGT-containing N-terminal region of human SARS-CoV-2 for evolutionary analysis. The results showed that there is a close genetic relationship between human SARS-CoV-2 and SARS-CoV, and some bat SARS-CoVs (Figure 4) on this region, except for the AIHVSGTNGTK fragment specifically in the SARS-CoV-2 spike glycoprotein. 

We further analyzed the homologous sites of the spike glycoproteins in human SARS-CoV-2 and SARS-CoV, as well as civet and bat SARS-CoVs. Among the homologous sites of the spike glycoprotein, only seven sites were found in both human SARS-CoV-2 and human SARS-CoV, which were different from the highly conserved sites (over 80%) in bat SARS-CoV (Figure S2 and Table S3). The eight identical sites include four in the spike receptor-binding domains, one in the coronavirus S2 glycoprotein peptide and three in the N-terminal domains. Importantly, a locus in the N-terminal domain of SARS-CoV-2 is similar to the counterpart in the human SARS virus but different from the loci found in more than 90% of bat SARS-like viruses. These results suggest that a prerequisite for a coronavirus to have potential for human infection is that it contains at least eight key homologous sites to bat SARS-CoV in its genome. Thus, the accumulation of mutant sites on those specific fragments of bat SARS-like viruses may effectively predict the next virus outbreak.

### 3.3. Analysis of Virus-HLA Binding Affinity for Allergenicity Assessment

A variety of pathogenic viruses can cause hypersensitivity reactions and are threats to human health. Therefore, it is of interest to investigate the allergenicity between virus proteins and host immune defenses. To assess the allergenicity and the infection potential of the virus to humans, we analyzed and classified HLA molecules that can tightly bind to the spike proteins of SARS-CoV-2 and SARS-CoV. Through the binding affinity analysis of human SARS-CoV-2 and SARS-CoV with HLA class I and II molecules, we found those HLA alleles that are predicted to bind to the spike protein from SARS-CoV-2 are very similar to those from SARS-CoV. The spike proteins from SARS-CoV-2, as well as SARS-CoV, are predicted to bind with high affinity to five human HLA class I alleles (Figure S3) and seven HLA class II molecules (Figure S4). These results suggest that human SARS-CoV-2 and SARS-CoV could induce similar strong immune responses in populations with the same genetic background. However, it is worth noting that the number and location of the HLA alleles with strong affinities for the spike proteins are not exactly the same for human SARS-CoV-2 and SARS-CoV, suggesting that actual immune responses to them may differ.

As listed in Table 1, the HLA class II alleles with a high binding affinity to SARS-CoV-2 include DRB3*03:01, DRB1*10:01, DRB1*09:01, etc. Among them, DRB3*03:01 is the most frequent allele, to which 79.59% of the fragments in S protein of SARS-CoV-2 can bind tightly (Table 1), indicating that patients with this allele may have severe immune responses after SARS-CoV-2 infection. It is worth noting that SARS-CoV also has peptides with a very high binding affinity to this HLA allele (Table S6). Certain HLA alleles are generally prevalent in the population. For example, the proportion of the HLA allele DRB1*09:01 is 24.28% in a population of 103,259 Chinese. These results demonstrate that SARS-CoV-2 is capable of causing severe immune response in most people with the above HLA class II alleles.


$$Ni=\overset{n}{\underset{i=1}{\sum }}Si*Qi*\left(2-Qi\right)$$

On the other hand, most of the other HLA alleles exhibited low or no binding affinity to SARS-CoV-2 and SARS-CoV. The frequency of DQB1*03:02, for example, is about 10.54% in the Chinese population (http://www.allelefrequencies.net, accessed on 27 August 2021). This allele has a low binding affinity to SARS-CoV-2. In addition, SARS-CoV-2 harbors far fewer peptides with high binding affinity to HLA class I alleles than class II alleles (Table 1 and Table S4). For example, the HLA I allele with the highest affinity to SARS-CoV-2, B*15:03, binds 97 peptides from SARS-CoV-2 with high affinity. Furthermore, the total proportion of high-affinity fragments for HLA I molecules in the total peptide fragments from SARS-CoV-2 is only 7.67%. Therefore, it would render SARS-Cov-2 more likely to induce the immune response through specific HLA class I/II molecules, resulting in distinct immune responses in different patients because of the genetic diversity of HLA genes.

### 3.4. Analysis of Meteorological Factors

**Figure 5:**
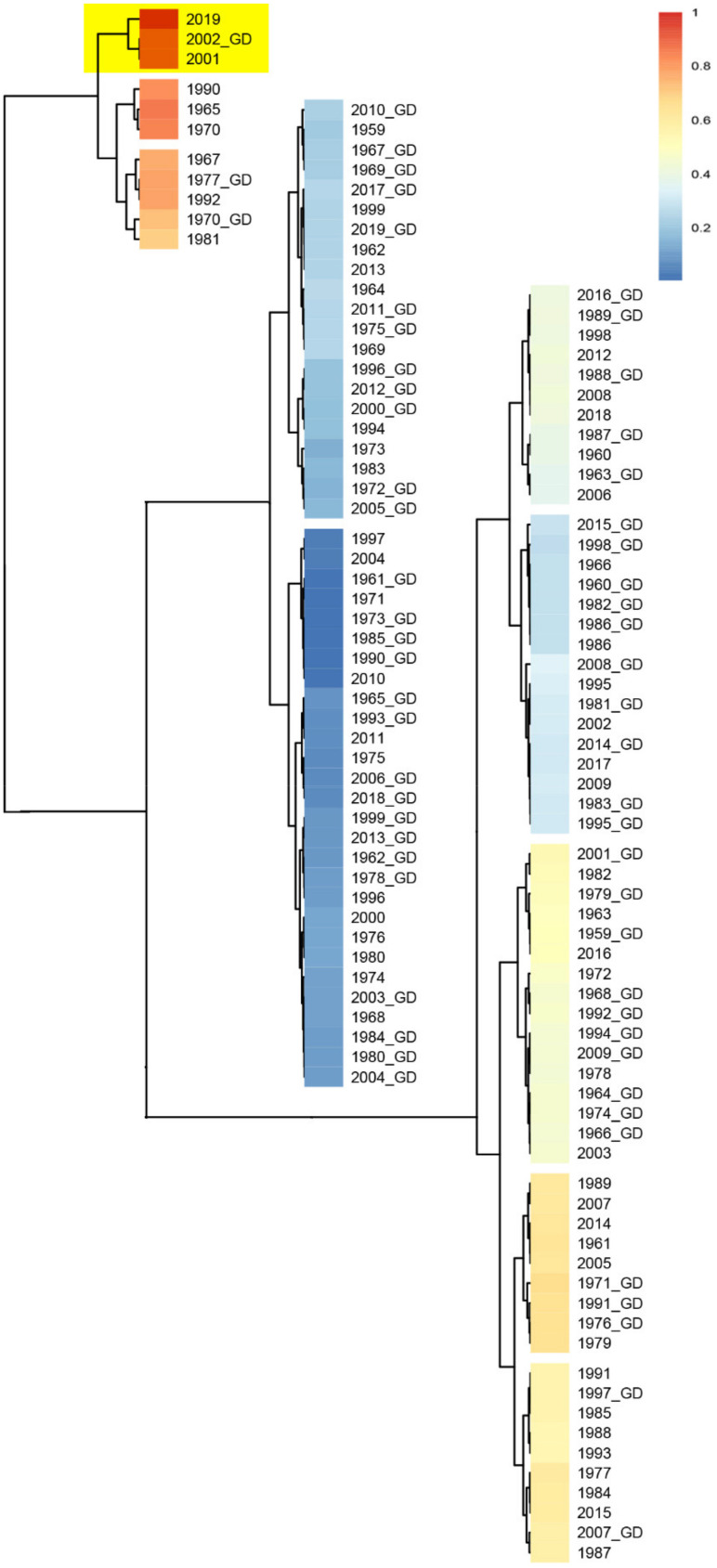
Correlation between Wuhan and Guangdong during 1951–2019 under the combined relative humidity characteristics. The correlation coefficient of each two years were calculated. G, the year of Guangdong. Cell color encodes correlation coefficients (red, positive correlation; blue, negative correlation). Color scale indicates the range of correlation coefficients. The correlation coefficient is assumed to be between 0 and 1, where 1 indicates the strongest possible association and 0 indicates the weakest possible association.

Strong allergenicity could be a causative agent of the virus. In addition, the effect of meteorological factors on viral transmission and outbreak at the host population have not yet been determined. It was reported that extreme meteorological factors can accelerate the mutation of viruses [9,10,29,30,31,32,33,34,35,36,37]. In this study, in order to find out the key climatic features of two coronavirus outbreaks in Wuhan and Guangdong in 2019 and 2002, respectively, we analyzed the climate data (mean temperature, maximum temperature, minimum temperature, precipitation, hours of sunshine, relative humidity) of Wuhan and Guangdong from 1951 to 2019. Based on 12 months and 6 climatic features, we constructed 257,985 combinations with different months and climatic features. Among all possible combinations between the climate factors, 406 combinations exhibited strong correlation between Wuhan 2019 and Guangdong 2002 (no strong correlation existed among other years). The frequency of occurrence and percentage of total combination of corresponding climate feature combinations in the 406 selected combinations are listed in Table S5. For example, precipitation appeared 158 times, which is the highest of all climatic combination features, accounting for 32.31% of the total combinations. In addition, 27.81% of the 406 selected combinations contained relative humidity alone, while 22.29% of them contained both relative humidity and precipitation, indicating that these are the key features of strong correlation between the climates of Wuhan in 2019 and Guangdong in 2002 (Figure 5). These extreme climate factors may accelerate the viral mutation rate, which could be one of the factors causing the virus outbreak.

**Figure 6:**
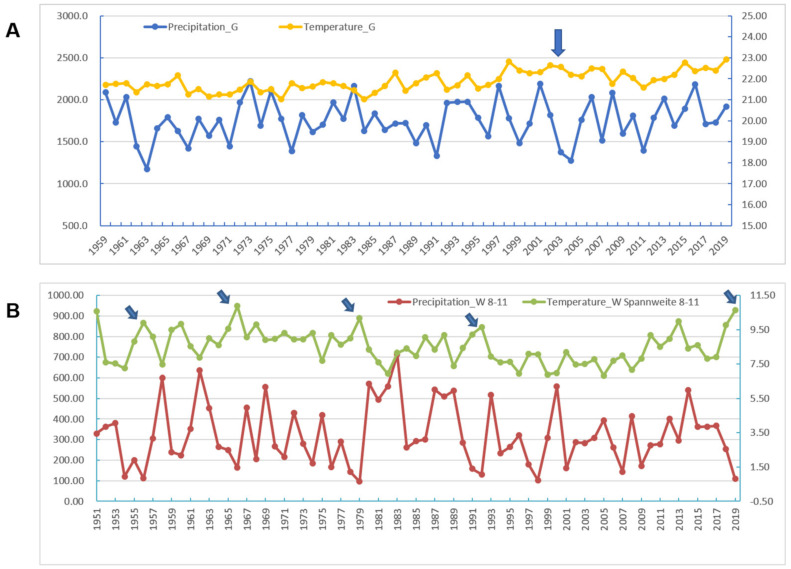
Analysis of meteorological factors in Guangdong and Wuhan in the years 1951 through 2019, where SARS and COVID-19 outbreaks occurred in 2002 and in 2019, respectively. (**A**). Annual precipitation and annual temperaTable 2002. (**B**). Distributions of the mean diurnal range of temperature and precipitation from August through November, during which in 2019, Wuhan experienced a severe drought in the summer and autumn seasons. The arrows point to extremely high temperatures and lower precipitation in years 1955, 1966, 1979, 1992 and especially in 2019. The extreme weather may favor viral mutation to more virulent forms.

According to a report from the Hubei Meteorological Bureau, the most serious drought in Hubei Province in the past 69 years occurred in the summer and autumn of 2019. During this abnormally long drought, much less precipitation was accompanied by hot weather. In general, a large fluctuation in temperature in Wuhan City between August and October was observed. When the maximum temperature, the minimum temperature, and the precipitation were used as the meteorological parameters from the August to October periods in the past 69 years, the results show that Wuhan 2019 was an independent branch of clustering with the actual climate characteristics (Figure S5). When the mean diurnal ranges of temperature and precipitation were used as parameters, the curves showed the extreme conditions in Wuhan in 2019 in terms of high temperatures and low humidity, similar to but more extreme than the temperature-precipitation relationship in Guangdong 2002–2003 (Figure 6).

## 4. Discussion

The goal of this study is to investigate the probable intermediate hosts and the allergenicity of the notorious virus SARS-CoV-2 to understand how this virus emerged. The phylogenetic analysis of the virus spike proteins indicates bats as a likely natural origin and rodents as the intermediate reservoir of SARS-CoV-2 and SARS-CoV. A variety of pathogenic viruses can cause hypersensitivity reactions and are threats to human health. Therefore, we evaluate the allergenicity between virus protein and host immune defenses. The results showed that both SARS-CoV-2 and SARS-CoV are predicted to bind to fourteen HLA class I and II molecules with super-high HLA allele-peptide affinities. Extreme climate might have promoted coronavirus to enable viral transmission and outbreak in the host population. Meteorological factors analysis shows that relative humidity and precipitation could be key factors causing the virus outbreak.

With the number of confirmed COVID-19 cases reaching 5,267,452 as of 24 May 2020, which is far more than the number of cases of severe acute respiratory syndrome (SARS), it is clear that the world is in the midst of a global pandemic. It is of the utmost importance to quickly discover the intermediate hosts of this virus and eradicate the source in order to prevent future outbreaks. Several research groups have recently attempted to address this issue [5,6,7,8]. Bats, minks, snakes, and pangolins, and many other creatures seemed to be possible candidates for the interspecies transfer of the novel virus from wildlife to humans, since these animals were sold as delicacies in this market. However, there are some challenging and unexplained facts. The first clinical cases published in *The Lancet* reported that >33% of the cases had no apparent link to the seafood market [2]. According to a report in the NEJM, although up to 84.5% of 1099 patients confirmed by laboratories had visited Wuhan city or had contact with Wuhan residents, only 1.9% of these patients had a history of direct contact with wildlife [38], which indicates a high potency of human-to-human transmission of this virus beyond the seafood market origin. Strikingly, of the 585 tested environmental samples, including 70 taken from the wildlife-trading shops and 515 collected from the COVID-19 patients served in shops and related blocks, 33 samples, 31 from the Western zone of the large market where wildlife was sold and 2 from other parts of the market, were positive for SARS-CoV-2 [39]. However, of the 31 positive samples, only 14 were derived from the wildlife-trading shops, whereas 19 positive samples were collected from other kinds of shops (https://3w.huanqiu.com/a/24d596/9CaKrnKp4T3?agt=8, accessed on 27 August 2021). Currently, it is unknown how the virus can be transferred directly from animal species to humans in the seafood market and how the virus could be spread among diverse foods in this market. This suggested that there may be animals that freely contact all kinds of foods and spread the virus everywhere in the market. 

The house mouse (*Mus musculus*) and Norway rats (*Rattus norvegicus*, also known as brown rats) are the most widely distributed and most successful mammals, except for humans, on the planet and have been commensal with humans for thousands of years [40]. These rodents prefer habitats proximate to human populations and thus are likely to be the intermediate hosts of the virus SARS-CoV-2. In some blocks of the Huanan Seafood Wholesale Market, animals are actively traded as delicacies, with their carcasses and viscera littering away day and night (https://tech.sina.com.cn/roll/2020-01-23/doc-iihnzhha4251798.shtml, accessed on 27 August 2021), thereby providing a food source for rats and mice. (https://tech.sina.com.cn/roll/2020-01-23/doc-iihnzhha4251798.shtml, accessed on 27 August 2021). The viruses harbored by wild animals would be therefore taken away by the foodies, thus rendering the viruses scattered everywhere in or even outside the Market and then transferred to humans.

In this regard, it is interesting that both SARS-CoV and SARS-CoV-2 possess dozens of fragments derived from rodents (rats and/or mice), respectively. Two fragments (EAEVQID/NHTSPDV) shared by both viruses are more conserved than other proteins encoded by the viruses. This explains why the two viruses cross-react with the antibodies generated against the other [41]. It also strongly suggests that these rodents might be the intermediate hosts of both SARS-CoV and SARS-CoV-2 transferred to humans. This assertion is corroborated by the following facts. Among the samples from the rats and mice captured in Guangzhou hospitals in 2003, 12.5% were SARS-CoV positive by anus swab tests and in these positive samples, 90%-96% exhibited sequence homology with SARS-CoV [42]. Moreover, around the Amoy Gardens housing complex in Hong Kong in 2003, SARS-CoV remnants were detected in four of the eight samples of rat droppings and in the throat or rectal swabs from at least one rat [43]. 

With regard to SARS-CoV-2, even though the Huanan Seafood Wholesale Market was shut down on 1 January 2020, animal carcasses and viscera were observed and living rats and mice were still present through 17–19 January when the High-level Experts Group of the National Health Commission arrived to investigate the outbreak. Similar to the SARS-CoV outbreak 18 years ago, it is possible that rats and/or mice acquired the SARS-CoV-2 virus from the viscera of butchered animals, including, for example, bats, minks, pangolins that served as natural reservoirs for the virus, when these animals were traded as delicacies in the Market. 

Two studies showed that SARS-CoV-2 has infected cat populations in Wuhan during the outbreak and argued that the virus was transmitted in cats [7,8]. This reinforces our proposal that mice and rats are the intermediate sources of SARS-CoV-2, since the rodents could eat many kinds of foods in the Market until they were eaten by cats. This conclusion could be strengthened if the SARS-CoV-2 viral sequence was found in rodents caught around that Market. 

Mice and humans have large-scale synteny across over 90% of their genomes but have a much lower extent of sequence orthology covering less than half of the two genomes [44]. Therefore, there are significant differences between the two species, especially within each of their MHC (major histocompatibility complex) genomic regions [45]. In this study, we calculated the binding affinity of the two virus spike proteins with human/mouse MHCs to deduce the allergenicity of the viruses, based on the danger theory [18,46] and the MHC restriction phenomenon [15,16,47,48]. Our results show that five human HLA class I alleles and nine human HLA class II alleles can bind tightly with the S protein fragments of SARS-CoV-2, accounting for 0.18% to 24.28% of the sampled Chinese populations (Table 1). Provided that the locus recombination frequencies of 2%-3% are negligible [49], the proportion of infection-susceptible individuals would be about 7.36% of the Chinese population. These data suggest that people who have these HLA genotypes would be severely affected by COVID-19 and develop obvious pathological symptoms if there were no intervention. The rate of refractory patients calculated from the epidemic data from 14 February to 21 March 2020 in China was 24.7% of the hospitalized patients (about 0.5% of whole Wuhan population), obviously different from the estimated value of 7.36% of the whole population. The discrepancy may result from the powerful non-medical and medical interventions that were implemented to control COVID-19 in China. By contrast, the remaining 92.64% of the population would be healthy without severe symptoms even if they were infected by SARS-CoV-2. Under all kinds of intervention, the proportion of asymptomatic population and paucisymptomatic cases would be much higher than 92.64%, as estimated by different researchers at different times to be 39.9–50.5% [50], 59% [51], or 90% [52] in the Chinese population. Taken together, specific patients who have been infected by SARS-Cov-2 may not have obvious symptoms, making prevention of COVID-19 incredibly challenging. The phenotypic frequencies in different countries are expected to be somewhat different. For example, the rate of refractory virus-susceptible individuals was estimated to be 4.78% of the American population without any interventions (Table 1).

**Figure 7:**
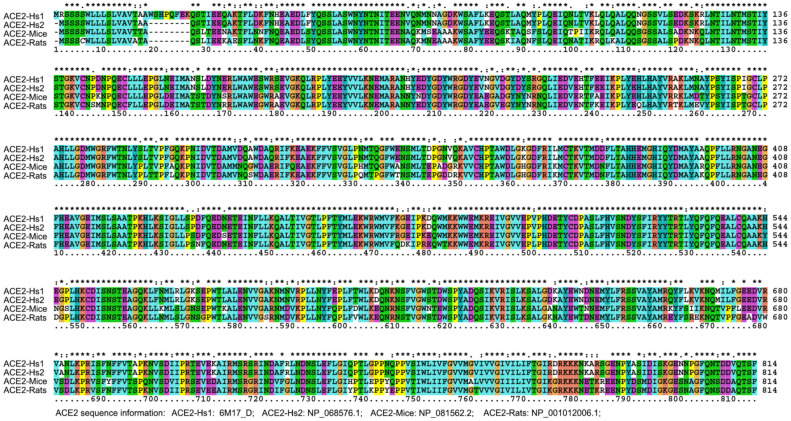
ACE2 Sequence alignment of humans, mice and rats.

According to sequence alignment with other SARS-CoVs, SARS-CoV-2 has an unusual insert of 10 amino acids (HVSGTNGTKR) in the N-terminal domain (Figure 3). This insert is aligned specifically to RaTK15, a SARS-like coronavirus reported to originate from bats *Rhinolophus affinis* (but not *Rhinolophus sinicus*), with 96.2% identity at the whole-genome level to SARS-CoV-2 [2]. No other animals have been reported with a higher sequence identity with the SARS-CoV-2. Ge et al. strongly suggested that Chinese horseshoe bats were the natural reservoirs of SARS-CoV, and that intermediate hosts may not be necessary for direct human infection by some bat SL-CoVs [53]. It is notable that Chinese horseshoe bats, *R. sinicus* and *R. affinis*, have a similar appearance, and *R. affinis* is the main variety of bat in the Hubei Province. Nevertheless, no SARS-CoV-like virus has been identified from the Hubei *R. affinis* bats, but from the bats *R. macrotis* and *R. ferrumequinum*, in which no viruses had been isolated by culture with Vero E6 cells from fecal swabs of the PCR-positive samples [54]. In addition, *R. pearsoni* bats are indigenous across the Yunnan province, China and Southeast Asia, and were suggested to harbor coronaviruses closely related to SARS that infected the human population [54]. The study [2] on viral infectivity into HeLa cells with or without the expressions of ACE2 proteins from human, Chinese horseshoe bats (*R. sinicus*, not *R. affinis*), civets, pigs, and mouses concluded that SARS-CoV-2 could use all but mouse ACE2 as an entry receptor in the ACE2-expressing cells; that is to say, the mouse ACE2 would not facilitate SARS-CoV-2 entry to mouse cells. This conclusion may be doubtful based on the homology analysis performed on ACE2. The alignment result indicated that the identity of ACE2 amino acid sequences between human and mice (*Mus musculus*) or rats (*Rattus norvegicus*) are 81.05% to 82.49% (Figure 7 and Table 2), respectively, which exceeds the threshold of greater than 70% sequence identities usually required to trigger cross-reactivity between proteins [55]. This empirical law supports that mouse ACE2 is a receptor for SARS-CoV-2. In fact, a previous study reported that SARS-CoVs can proliferate in the mouse without severe symptoms [56]. Even if the above mouse ACE2 assays [2] were correct, an alternative route involving the CD147-spike protein would also help SARS-CoV-2 to invade host cells [57], further explaining the above conflicting results and supporting the rodents as a potential intermediate reservoir of SARS-CoV-2. That is to say, viral proliferation in rodents can be maintained without symptoms because MHC alleles in rodents have no ultrahigh binding affinity to proteins from SARS-CoV-2 or SARS-CoV (data not shown). It is therefore tempting to deduce that the mouse could be a long-term host of human SARS-CoV-2. Furthermore, according to our previous analysis, after a cross-species jump in 1991 and a human-adapted strain formed in 1998, SARS-CoV may still exist in humans (https://arxiv.org/abs/1305.2659, accessed on 27 August 2021). Therefore, the entry receptor ACE2 is not a problem for the coronavirus to attack humans from then on, no matter whether RaTK15 was isolated from *R. pearsoni*, *R. sinicus*, or *R. affinis*. 

SARS-CoV-2 is a positive-sense, single-stranded RNA coronavirus. It possesses a large RNA genome and undergoes RNA recombination, as in other coronaviruses, at a high frequency of nearly 25% for the entire genome [58], thus driving frequent species-transmission adaptation. Another report suggested that SARS-CoVs were likely caused by mutations and natural selection in addition to recombination [59]. Moreover, an average female rodent gives birth approximately seven times per year, which would lead to much a higher rate of mutations of the viruses maintained, compared to a deduced general mutation rate of 0.80~2.38 × 10^−3^ nucleotide substitutions per site per year for SARS-CoV [60]. Furthermore, during the August through November period in 2019, the most serious drought and highest temperatures in the summer and autumn time frame were experienced in Wuhan in the past 68 years (Figure 6). This climate could have provided favorable conditions for virus mutation from a mild form to the highly virulent SARS-CoV-2. Based on the causality triangle of viruses, hosts and environmental conditions, even if the nucleotide fragments of an intermediate host were integrated into the virus, those fragments could not easily be detected because of RNA recombination. Since cross-species transmissibility depends on protein functions, the amino acid sequences of the virus can provide compelling evidence to support identification of intermediate hosts.

Interestingly, both SARS-CoV and SARS-CoV-2 are inherently capable of reacting with different allelic forms of HLA molecules and tightly binding dozens of different HLA molecules. This means that these two viruses would have similar allergenicity and would trigger similar pathophysiological insults in humans. This is supported by autopsy and biopsies of cadavers of patients who died from SARS-CoV-2, because the pathological characteristics of COVID-19 strongly resemble those seen in SARS and Middle Eastern respiratory syndrome (MERS) coronavirus infections [61,62,63]. However, the cytokine-based endotypes of critically ill COVID-19 patients who are insensitive to treatment with steroids because of an increased concentration of the highly proinflammatory cytokine IL-17A produced by CCR4^+^CCR6^+^ Th17 in CD4^+^ T cells [63,64] would be quite different from those of SARS patients for whom steroid treatment is beneficial because of the increased presence of type 2 cytokines [65,66]. Therefore, clinical treatments for SARS-CoV-2 patients will be different from those employed on SARS patients. On the other hand, unlike human HLA, mouse MHC does not bind strongly to the spike protein of SARS-CoV-2, suggesting that there would be no symptoms when rodents become infected with this virus. In fact, although SARS-CoV can replicate in the lungs of young mice following infection, such mice do not harbor replicated SARS-CoV in both lung and intestinal tissue and they do not show signs of illness. These mice present either subclinical infection or very mild disease after simultaneous inoculation intranasally and orally [56,67]. Therefore, it is likely that these rodents would not become ill in response to SARS-CoV-2 infection even if they harbored this virus. This situation provides conditions for the spread of the virus in humans and the rodents until herd immunity develops in the two populations. This is corroborated by our previous research data showing that SARS-CoV may still exist in humans (https://arxiv.org/abs/1305.2659, accessed on 27 August 2021). 

It is thus likely that a SARS epidemic could recur when the meteorological conditions in the world are suitable for SARS-CoV-2 mutation. The virus would be maintained in general populations who have no high binding-affinity HLA alleles and be transferred between individuals. As described above, more than 92.64% of the population harbors the virus with no obvious symptoms, meaning that many people will be SARS-CoV-2 positive as detected by nucleic acid testing. We have demonstrated that weakly virulent SARS-CoVs might still exist in humans for years (https://arxiv.org/abs/1305.2659, accessed on 27 August 2021). These existing SARS-CoVs have significant potential to evolve into highly virulent strains when favorable meteorological conditions occur, highlighting the potential risk for reemergence of SARS as well. Based on the mutation rate of coronavirus and meteorological extremes occurring because of climate change, we speculate that SARS could re-emerge in the near future in a new form. A SARS vaccine is therefore urgently needed. However, a SARS-like chimeric virus experiment demonstrated that both monoclonal antibody and vaccine approaches and prophylactic modalities failed to neutralize and protect from infection by those CoVs that possess a novel spike protein [68]. Advanced strategies and regimens will need to be developed. For example, a novel vaccine against a pool of the most highly virulent mutant strains could be prepared in advance.

**Hypothesis** **1.** *Self-limitation and spontaneous mutation within the virus-infected population.*

With these results, we therein proposed a hypothesis for the future course of the coronaviruses. When a virus mutates into a novel one and severely infects one (or several) group(s) of individuals with specific MHC genotype(s), these susceptible individuals either die or heal with the development of immunity. The vast majority of individuals who range from being paucisymptomatic to asymptomatic or having recovered from the disease still harbor the novel virus while it is spread within the population with no epidemic. When conditions favor mutations, the virus becomes more virulent and targets individuals harboring other type(s) of MHC genotype(s), leading to soaring infection numbers and another epidemic. A new cycle would therefore start within the population. When the virus has acquired mutations favoring a cross-species jump, increasing infection numbers would lead to an epidemic followed by asymptomatic transmission within the new species into which the virus has jumped. This phenomenon will occur in many different mammals including humans, bats, rodents, etc. Furthermore, when the virus accumulates the requisite mutations enabling interspecies transmission and binding of all MHC alleles among another species population with high affinity, a much deadlier super virus could emerge to eradicate the species. The only way to defend against such a super virus is to employ prophylactic modalities, such as early immunization with a hypoallergenic virus that has been gradually attenuated from the super virus, as exemplified by the incidence of smallpox versus cowpox.

In summary, our results indicate that both SARS-CoV-2 and SARS-CoV are naturally originated from bats and might be transmitted to humans through rodents. This was demonstrated by carrying out comprehensive amino acid sequence analysis and comparison of sliding sequence fragments of the novel virus with all sequences from mammals available in the NCBI database. SARS-CoV-2 and SARS-CoV have similar binding affinities to the HLA antigen and would have similar potential to induce inflammation. Different populations have distinct allele distribution patterns and thus variable infection rates. It is predicted that the virus will severely infect 4.78% to 7.36% of the American and Chinese populations, respectively, and would make them suffer severe symptoms. Meteorological factor analysis indicates that Wuhan 2019 and Guangdong 2002–2003 have similar climate features, with extremely high temperatures and exceptionally low precipitation, which might imply some link between the climate environment and the survival and development of the coronaviruses. Early immunization with allergenically-engineered virus together with a continued surveillance of meteorological factors and viral mutations may serve as one of the most powerful prophylactic modalities to fight this virus. 

## Figures and Tables

**Table 1:** Specific HLA alleles and high affinity peptides from SARS-CoV-2 spike protein.

HLA Class.	Alleles	No. of High Affinity Peptides	% of High Affinity Peptides	No. of Ultrahigh Affinity Peptides	% of Individuals That Have the Specific HLA Allele in the Population of	
					Chinese	American
I	B*15:03	97	7.67	**1**	0.18	2.69
I	A*02:03	52	4.11	**1**	8.12	1.07
I	B*15:17	51	4.03	**3**	0.81	0.90
I	A*24:03	35	2.77	**2**	0.43	0.57
I	A*30:01	26	2.06	**1**	14.27	/
II	DRB3*03:01	998	79.59	**64**	/	12.72
II	DRB1*10:01	805	64.19	**30**	2.75	3.41
II	DRB1*09:01	555	44.26	**9**	24.28	4.94
II	DRB1*16:02	528	42.11	**14**	5.15	1.96
II	DRB1*13:02	481	38.36	**38**	7.58	9.56
II	DRB1*01:01	375	29.90	**8**	4.14	10.75
II	DRB1*11:01	175	13.96	**2**	8.49	/

Affinity over 0.5 was defined as “high affinity”, therefore, only peptides with affinity over 0.5 were accounted for high affinity peptides. The percentage of high affinity peptides is calculated as total high affinity peptides divided by total peptides that the spike protein harbors and the specific HLA molecule can bind, multiplied by 100. Affinity 0.9 (HLA Class I) and affinity 0.8 (HLA Class II), the top affinities of the panallergen profilin Q64LH0, were defined as the ultrahigh affinity cutoff values. All the original allele frequency and corresponding sample data were downloaded from the website (http://www.allelefrequencies.net/), where the frequency of DRB3*03:01 is unavailable for Chinese population, and those of A*30:01 and DRB1*11:01 are unavailable for American population, DRB5*01:01 and DRB3*02:02 (not shown) unavailable for both Chinese and American populations. The percentage of individuals that have the alleles in the sampled subpopulations was calculated based on sample sizes and allele frequencies. According to the Hardy-Weinberg Equilibrium, the number (***Ni***) of individuals that have the allele in the total of the sampled ***i*** subpopulation was roughly calculated based on the sample sizes (***Si***) and allele frequencies (***Qi***) through the following formula. The larger the subpopulation, the more accurate the ***Ni*** result.

**Table 2:** Amino acid sequence comparison of human ACE2 with those from rat and mouse.

ACE2 Comparison	Max Score	Query Coverage	E Value	% of Identities
ACE2-Hs1 vs. ACE2-Hs2	1673	99%	0	99.01
ACE2-Hs1 vs. ACE2-mouse	1361	98%	0	81.05
ACE2-Hs1 vs. ACE2-rat	1353	96%	0	82.49
ACE2-Hs2 vs. ACE2-mouse	1369	98%	0	81.86
ACE2-Hs2 vs. ACE2-rat	1360	98%	0	82.37

ACE2 sequence information: **ACE2-Hs1**, ACE2 6M17_D from *Human sapien*. **ACE2-Hs2**, NP_068576.1 from *Human sapien*. **ACE2-mouse**, NP_081562.2 from *Mus musculus*. **ACE2-rat**, NP_001012006.1 from *Rattus norvegicus*.

## Supplementary Materials

The following are available online at www.mdpi.com/article/10.3390/healthcare9091132/s1, Figure S1. NCBI BLAST results (20200121) of HAIHVSGT, a murine specific peptide in the SARS-CoV-2 Spike glycoprotein using the NCBI reference protein library, Figure S2. The key sites for bat SARS-like virus infecting human. The key positions in the three functional regions of the spike glycoprotein (N-terminal domain, receptor binding domain, and Coronavirus S2 glycoprotein), and the amino acid distribution in human SARS-CoV and bat SARS-CoV are presented, Figure S3. Analysis of HLA class I molecular binding affinity with SARS-CoV-2 and SARS-CoV. HLA class I molecular were divided into two groups: high binding capacity and low binding capacity. For each molecular, the binding affinity with four different functional segments (marked with different colors) of spike glycoprotein were presented, Figure S4. Analysis of HLA class II molecular binding affinity with SARS-CoV-2 and SARS-CoV. HLA class II molecular were divided into two groups: high binding capacity and low binding capacity. For each molecular, the binding affinity with four different functional segments (marked with different colors) of Spike glycoprotein, Figure S5. Correlation between specific climate characteristics including maximum temperature, minimum temperature and precipitation in Wuhan from 1951 to 2019. Cell color encodes correlation coefficients (Red, positive correlation; Blue, negative correlation). Color scale indicates the range of correlation coefficients. The correlation coefficient is assumed to be between 0 and 1, where 1 indicates the strongest possible associations and 0 indicates the weakest possible association. Table S1. Similarity screening of SARS-CoV-2 Spike glycoprotein in protein database were presented, Table S2. Similarity screening of SARS-CoV Spike glycoprotein peptides in protein database, Table S3. The pivotal loci for bat SARS-like viruses to infect humans, Table S4. Binding affinity of HLA Class I and II molecules with SARS-CoV spike protein, Table S5. The sample size and risk individuals of HLA Class I/II in Chinese and American, Table S6. Correlation analysis of climatic feature combinations between Wuhan 2019 and Guangzhou 2002.
