# Supplementary material for: SARS-CoV-2: Origin, Intermediate Host and Allergenicity Features and Hypotheses
Source: Healthcare (Basel). 2021 Aug 30;9(9):1132. doi: 10.3390/healthcare9091132 (PMC8466535; doi:10.3390/healthcare9091132)
Supplement: Supplementary file 1 [file healthcare-09-01132-s001.zip › healthcare-1018300-supplementary.pdf]

Figure S1-5

Description	Max Score	Total Score	Query Cover	E value	Per. Ident	Accession
peptidase S8 [Bacteroidetes bacterium 4572_77]	27.8	27.8	100%	132	100.00%	OYT15603.1
hypothetical protein SARC_03566 [Sphaeroforma arctica JP610]	27.8	27.8	100%	132	100.00%	XP_014158107.1
PDZ and LIM domain protein 7 isoform X3 [Mus musculus]	27.8	27.8	100%	134	100.00%	XP_006517412.1
PDZ and LIM domain 7, isoform CRA_b, partial [Rattus norvegicus]	27.8	27.8	100%	137	100.00%	EDL93973.1

Figure S1. NCBI BLAST results (20200121) of HAIHVSGT, a murine specific peptide in the SARS-CoV-2 Spike glycoprotein using the NCBI reference protein library.

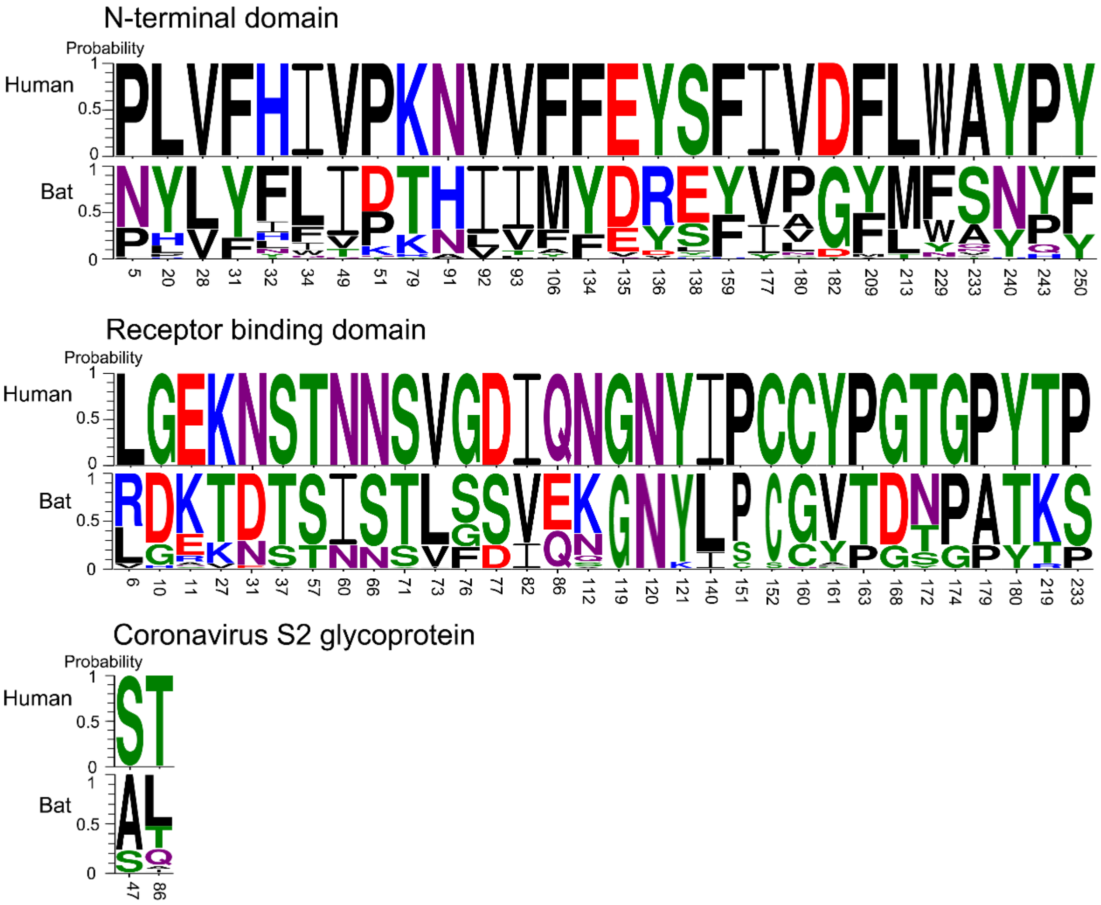

Figure S2. The key sites for bat SARS-like virus infecting human. The key positions in the three functional regions of the spike glycoprotein (N-terminal domain, receptor binding domain, and Coronavirus S2 glycoprotein), and the amino acid distribution in human SARS-CoV and bat SARS-CoV are presented.

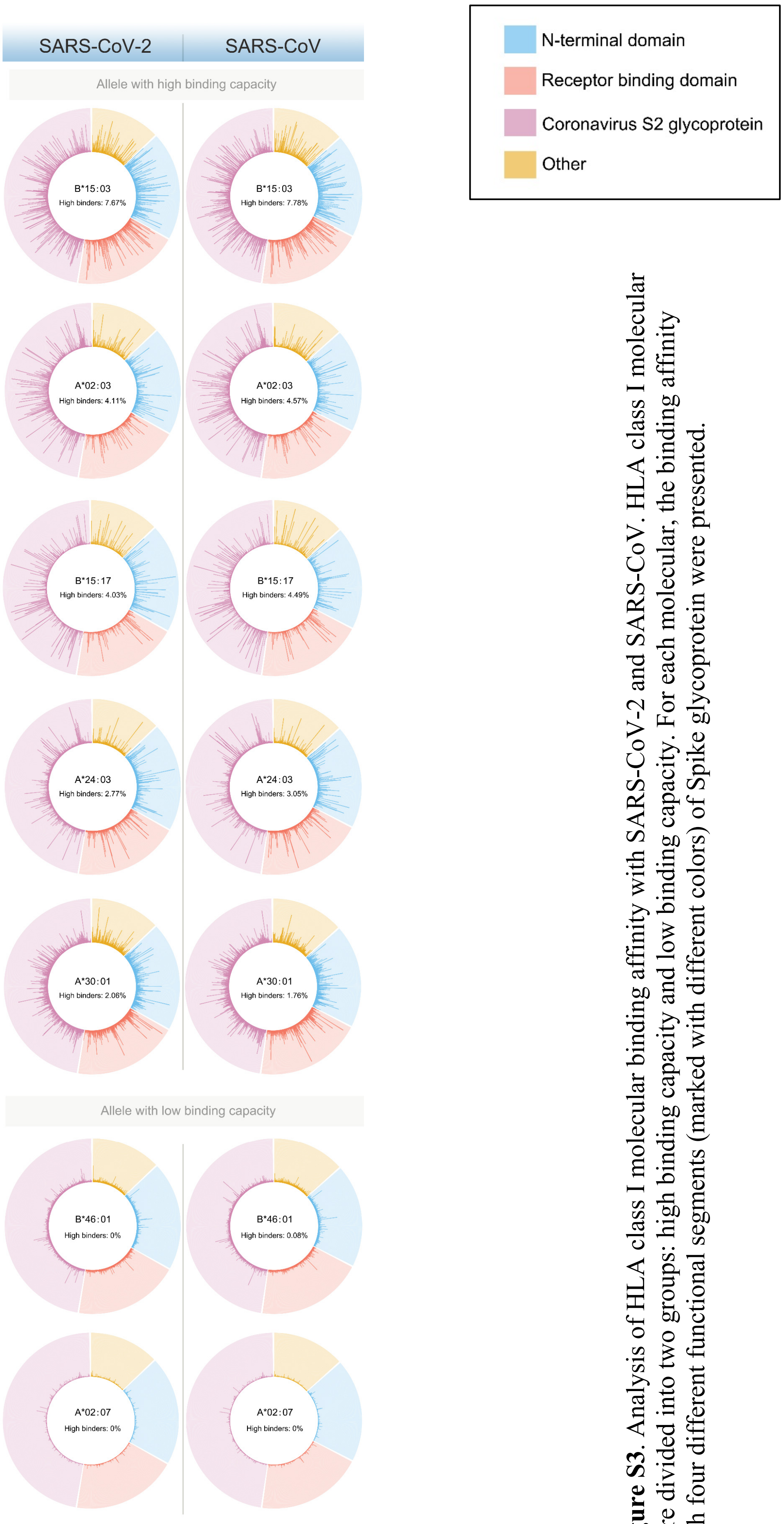

Figure S3. Analysis of HLA class I molecular binding affinity with SARS-CoV-2 and SARS-CoV. HLA class I molecular were divided into two groups: high binding capacity and low binding capacity. For each molecular, the binding affinity with four different functional segments (marked with different colors) of Spike glycoprotein were presented.

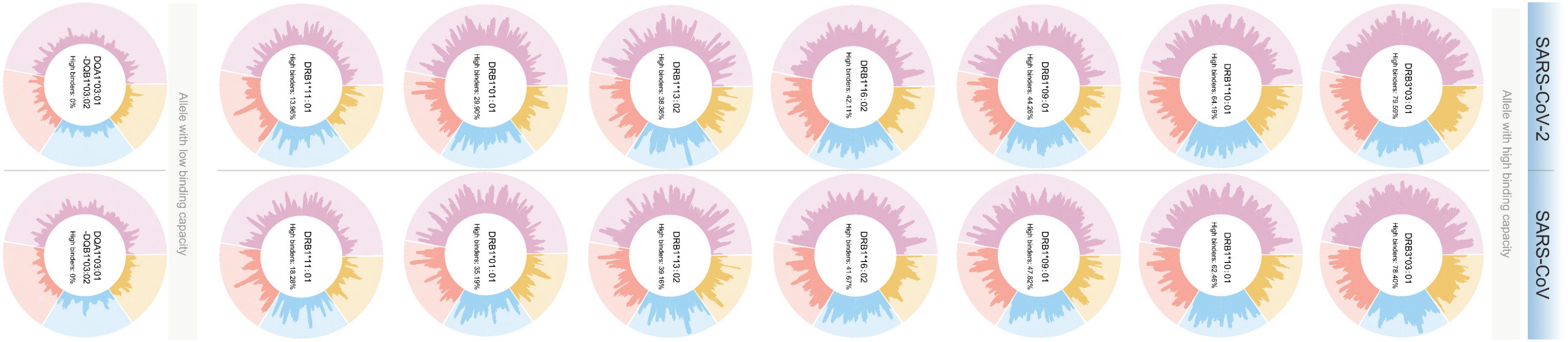

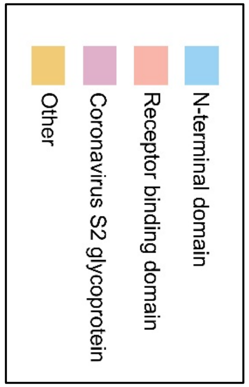

Figure S4. Analysis of HLA class II molecular binding affinity with SARS-CoV-2 and SARS-CoV. HLA class II molecular were divided into two groups: high binding capacity and low binding capacity. For each molecular, the binding affinity with four different functional segments (marked with different colors) of Spike glycoprotein were presented.

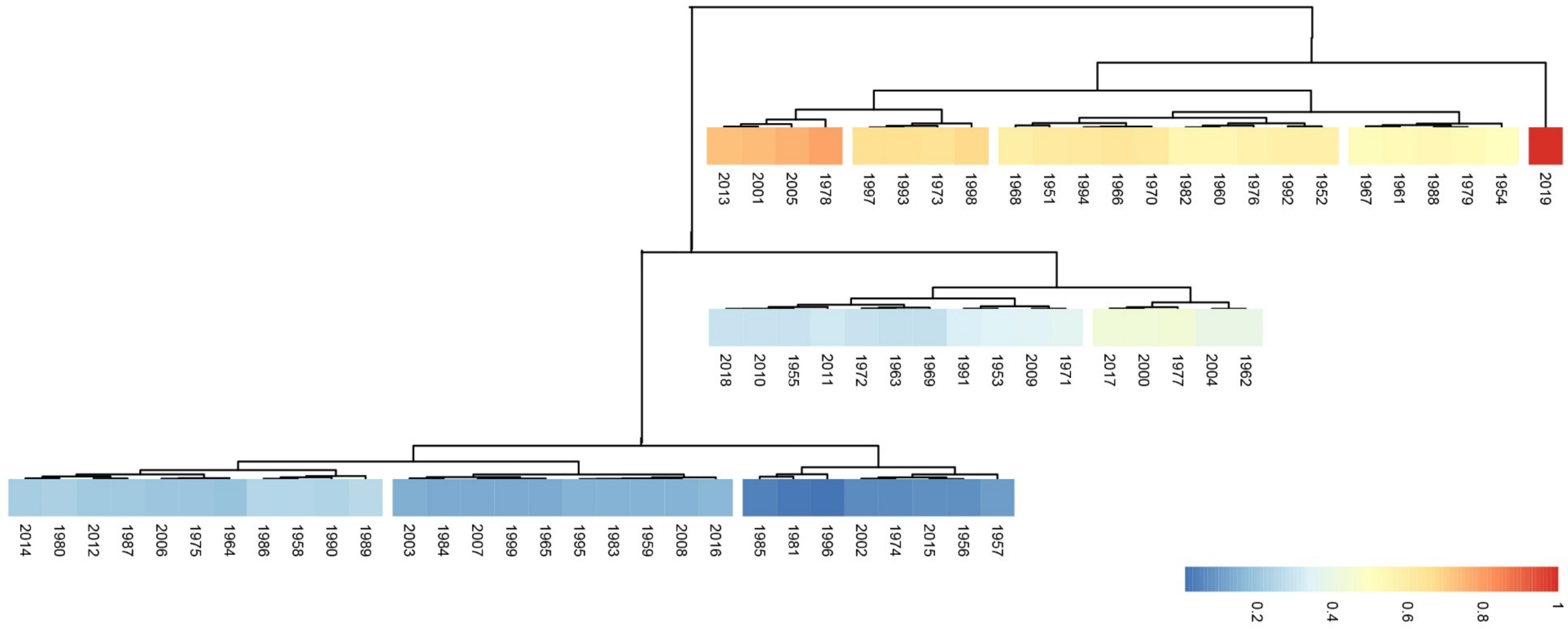

Figure S5. Correlation between specific climate characteristics including maximum temperature, minimum temperature and precipitation in Wuhan from 1951 to 2019. Cell color encodes correlation coefficients (Red, positive correlation; Blue, negative correlation). Color scale indicates the range of correlation coefficients. The correlation coefficient is assumed to be between 0 and 1, where 1 indicates the strongest possible associations and 0 indicates the weakest possible association.

Table S1-6

Table S1. Similarity screening of SARS-CoV-2 Spike glycoprotein in protein database

Peptide	Distribution	No. of matched sequences from all species	No. of matched mouse sequences	Proportion of mouse sequences
***EAEVQID**	S2 domain	18	11	61.11%
*NCTEVPVA	/	14	8	57.14%
*TMSLGAE	/	4	1	25.00%
LKGCCSC	/	17	4	23.53%
TQRNFY	/	50	7	14.00%
*HAIHVSGT	N-terminal domain	8	1	12.50%
TLLALHR	/	52	6	11.54%
***NHTSPDV**	S2 domain	26	3	11.54%
*DSFVIRGD	Receptor binding domains	57	5	8.77%
*ELLHAPA	Receptor binding domains	12	1	8.33%
RKSNLKP	/	46	2	4.35%
FKNLREF	/	182	5	2.75%
EIDRLNE	/	39	1	2.56%
DLEGKQG	/	40	1	2.50%
VYSTGSN	/	90	2	2.22%
SNIIRGW	/	247	j4	1.62%
TWRVYST	/	143	1	0.70%

First row, Peptide fragments from the spike protein of SARS-CoV-2. Mouse specific peptides were marked with asterisks; peptides commonly shared both by SARS-CoV-2 and SARS-CoV were marked in boldface. The number of matched peptides in different species were the retrieved entry number of similarity screening in the protein database.

Table S2. Similarity screening of SARS-CoV Spike glycoprotein peptides in protein database

Peptide	Distribution	No. of matched sequences from all species	No. of matched mouse sequences	Proportion of mouse sequence s
***EAEVQID**	S2 domain	18	11	61.11%
*QTQAGCL	/	10	5	50.00%
*LTDDMIA	S2 domain	17	6	35.29%
NKSQSVI	/	5	1	20.00%
***NHTSPDV**	S2 domain	26	3	11.54%
FYSNVTG	/	33	2	6.06%
NQKQIAN	/	73	4	5.48%
VNCTDVS	/	64	3	4.69%
ASYHTVS	/	250	10	4.00%
FLLFLTL	/	102	4	3.92%
NKAISQI	/	26	1	3.85%
EIDRLNE	/	39	1	2.56%
ILTAFSP	/	115	1	0.87%
VYAWERK	/	236	1	0.42%
YLRHGKL	/	250	1	0.40%

First row, Peptide fragments from the spike protein of SARS-CoV-2. Mouse specific peptides were marked with asterisks; peptides commonly shared both by SARS-CoV-2 and SARS-CoV were marked in boldface. The number of matched peptides in different species were analyzed through similarity screening in the protein database.

Table S3. The pivotal loci for bat SARS-like viruses to infect humans

Spike glycoprotein	Sequence position	SARS-CoV-2	Human SARS	Over 80% Bat SARS-like
N-terminal domain	31	F	F	Y
	182	D	D	G*
Receptor binding domain	66	N	N	S
	119	G	G	-
	140	I	I	L
	233	P	P	S
Coronavirus S2 glycoprotein	47	S	S	A

The Spike glycoprotein from SARS-Cov-2, human SARS and Bat SARS were homology analyzed to screen out the conserved key sites that consistent between human SARS-CoV-2 and human SARS-CoV, but different from the highly conserved sites (over 80%) in bat SARS-Cov. The conserved amino acids and their sequence position were listed in Line 2-5; -, missed site in bat SARS-CoV; *, the site is over 90% conserved in bat SARS-CoV.

Table S4. Binding affinity of HLA Class I and II molecules with SARS-CoV spike protein.

HLA Class	Alleles	No. of high affinity peptide
I	B*15:03	97
I	A*02:03	57
I	B*15:17	56
I	A*24:03	38
I	A*30:01	22
II	DRB3*03:01	969
II	DRB1*10:01	772
II	DRB1*09:01	591
II	DRB1*16:02	515
II	DRB1*13:02	484
II	DRB1*01:01	435
II	DRB1*11:01	226

Affinity over 0.5 was defined as High affinity; Affinity over 0.9 (HLA Class I) or 0.8 (HLA Class II) was defined as ultrahigh affinity based on the top affinities of the panallergen profilin Q64LH0; Only peptides with affinity over 0.5 in total peptides were calculated.

Table S5. The sample size and risk individuals of HLA Class I/II in Chinese and American

HLA Class	Alleles	Sample Size		Risk Individuals	
		Chinese	American	Chinese	American
I	B*15:03	6491	2907846	11.36	78334.76
I	A*30:01	14598	/	2082.90	/
I	B*15:17	7088	3160803	57.37	28456.36
I	A*02:03	8317	2964487	675.63	31655.43
I	A*24:03	6724	2906108	28.59	16469.68
II	DRB3*03:01	/	2670687	/	339605.84
II	DRB1*10:01	9131	2910443	250.97	99216.22
II	DRB1*01:01	8961	3039733	371.01	326878.36
II	DRB1*11:01	9131	/	250.97	/
II	DRB1*09:01	9761	2915687	2369.80	144096.06
II	DRB1*16:02	9788	3042254	503.91	59775.92
II	DRB1*13:02	9285	2977351	703.63	284489.21
	TOTAL	99275	29495399	7306.14	1408977.84
	Ratio	/	/	0.0736	0.0478

Table S6. Correlation analysis of climatic feature combinations between Wuhan 2019 and Guangzhou 2002

Climate feature combinations	Frequency of combination occurrences	Percentage of occurrence in total combinations
Precipitation	158	32.31%
Relative humidity	136	27.81%
Relative humidity + Precipitation	109	22.29%
Minimum temperature + Precipitation	1	0.20%
Maximum temperature +Precipitation	1	0.20%
Minimum temperature	1	0.20%

There are 406 combinations have a strong correlation between Wuhan 2019 and Guangdong 2002 (no strong correlation existed among other years). The frequency of occurrence and percentage of total combination of corresponding climate feature combinations in 406 eligible combinations were listed.
